# Biological Activities of Novel Kombuchas Based on Alternative Ingredients to Replace Tea Leaves

**DOI:** 10.3390/ph18111722

**Published:** 2025-11-13

**Authors:** Noemi Hontana-Moreno, Diego Morales

**Affiliations:** Departmental Section of Galenic Pharmacy and Food Technology, Veterinary Faculty, Complutense University of Madrid, Av. Puerta del Hierro, s/n, 28040 Madrid, Spain; nhontana@ucm.es

**Keywords:** kombucha, SCOBY, antioxidant, phenolic compounds, SWOT, fermentation

## Abstract

**Background/Objectives**: Traditional kombucha is produced by fermenting a sweetened infusion of *Camellia sinensis* leaves with a symbiotic consortium of bacteria and yeasts (SCOBY). The growing interest in this beverage has driven the exploration of alternative substrates, including a wide range of plant-based raw materials, such as leaves, fruits, flowers, and seeds. Consequently, numerous products are being investigated for their differential properties, not only organoleptic but also nutritional and bioactive. This review aims to summarize recent advances in alternative kombucha research, focusing on the substrates used, their physicochemical and biochemical characteristics, and the biological activities studied. **Methods**: A comprehensive literature search was conducted to select articles related to alternative kombuchas. A critical analysis of their current state was carried out through the Strengths, Weaknesses, Opportunities, and Threats (SWOT) methodology. **Results**: The SWOT analysis led to the identification of strengths, including promising in vitro results and growing consumer interest; weaknesses, including a lack of animal studies, clinical trials, and approved health claims, and an excessive focus on antioxidant activity and phenolic compounds; opportunities, including substrate diversity, innovation, and consumer education; and threats, including elaboration risks, misinformation, competitors, and potential consumer rejection. **Conclusions**: Despite the promising results achieved to date, it is essential that the scientific community and the food industry continue efforts to generate robust evidence, particularly through clinical validation, in order to draw reliable conclusions regarding the benefits of alternative kombuchas for human health.

## 1. Introduction

Kombucha is a fermented beverage traditionally prepared by fermenting sweetened tea leaves with a symbiotic consortium of bacteria and yeast (SCOBY). Its origins are somewhat uncertain, but historical records suggest that kombucha (or analogous beverages) were consumed in East Asia, particularly in China, at least 2200 years ago, and later spread to Japan, Russia, and Eastern Europe. However, the popularity of this millenary drink in Western countries was significantly delayed until the 20th century [1,2]. Conventionally, kombucha is made using *Camellia sinensis* leaves, sugar (commonly sucrose), and hot water; once the infusion has cooled to room temperature, the SCOBY is added [3]. This complex microbial consortium includes key yeast genera, such as *Saccharomyces*, *Zygosaccharomyces*, *Kluyveromyces*, *Dekkera*, etc.; acetic acid bacteria (AAB) genera, such as *Acetobacter*, *Gluconobacter,* and *Komagataeibacter*; and lactic acid bacteria (LAB) genera, such as *Lactobacillus*, *Lactococcus,* and *Oenococcus* [4,5].

During fermentation, yeasts, through the action of the enzyme invertase, hydrolyze sucrose into glucose and fructose, which are subsequently fermented to produce ethanol. In turn, AAB oxidize glucose to gluconic and glucuronic acids and utilize ethanol to generate acetic acid. They also polymerize cellulose chains, giving rise to the characteristic structures of the SCOBY, with species such as *Komagataeibacter xylinus* being particularly active in this process. In addition, LAB contribute by producing lactic acid [6,7].

The fermentation time of kombucha varies according to the industrial purpose, typically ranging from 7 to 10 days at temperatures between 20 and 25 °C. Over the course of the process, various physicochemical and chemical changes occur. As expected, the production of organic acids and their release from raw materials lower pH values, thereby altering the overall chemical composition. The commonly accepted safe pH range is between 2.5 and 4.2. Values below this range indicate excessive acidity, which may compromise sensory attributes and consumer health, whereas values above it increase the risk of growth of undesirable microorganisms [8]. Moreover, sugar concentrations also decline throughout the fermentative process; ethanol is first produced by yeasts and then partially or totally oxidized by AAB, and other compounds, such as proteins, peptides, and phenolic and volatile compounds, may be altered by microbial metabolism, binding, degradation, or precipitation phenomena. Collectively, these changes influence flavor, aroma, safety, and potential bioactivity [7,8,9].

Regarding this mentioned bioactivity, one of the key attractions of kombucha is its content of biologically active compounds. These include phenolic compounds (flavonoids, catechins, and tannins), organic acids (acetic, gluconic, and glucuronic), vitamins (C and B groups), minerals, peptides, and specific molecules, such as D-saccharide-1,4-lactone (DSL) [10,11]. In vitro studies have documented antioxidant, anti-inflammatory, hypoglycemic, and antitumoral activities, among others, for tea kombucha. However, there is a relative paucity of in vivo research, especially of clinical trials in humans [11]. To date, only a few human studies have been reported, often with small sample sizes and limited in duration, scope, and consistency of the kombucha formulations used. Thus, strong conclusions about efficacy, dosage, or mechanisms in humans remain underdeveloped. In fact, a recent systematic review found eight clinical trials with durations ranging from 10 days to 10 weeks, concluding that kombucha consumption may alleviate gastrointestinal symptoms and demonstrates modest capacity as a gut and salivary microbiota modulator, but it needs further robust research to confirm these promising effects since available clinical trial data are still limited and heterogeneous [12].

As previously mentioned, conventional kombucha is prepared using *C. sinensis* leaves, mostly green and black varieties. However, over the past few decades, extensive research within both the food industry and academia has explored alternative substrates for its production. While animal-based matrices, such as milk, as well as fungal sources, like mushrooms and truffles, and even algae, have been investigated, plant-based substrates remain the most commonly employed substitutes for tea leaves. These include fruits, herbs, flowers, and various by-products, which are primarily selected to impart distinctive sensory attributes and to enhance the bioactive composition of the beverage, thereby contributing to its potential functional value [13].

The present review aims to provide a comprehensive overview of recent advances in the development of kombucha produced from substrates other than traditional tea. Particular attention is given to their physicochemical and biochemical features, as well as to the biological activities that have been reported and the experimental models used to evaluate them. In addition, this review seeks to critically assess the current state of knowledge on alternative kombuchas, employing the SWOT (Strengths, Weaknesses, Opportunities, and Threats) framework, in order to identify research gaps, methodological limitations, risks, and promising directions for future work.

## 2. Results and Discussion

### 2.1. Alternative Substrates to Replace Tea in Innovative Kombucha Beverages

As was indicated in Section 1, traditional kombucha is prepared using tea leaf infusions as a complementary carbohydrate source together with sugarcane, also providing valuable compounds such as organic acids, vitamins, minerals, peptides, pigments, and phenolic compounds [7]. Black and green teas are the most used varieties in conventional kombucha, although all *C. sinensis* types, including oolong, white, or yellow tea, can also be employed [14]. Nevertheless, during the last decades, alternative substrates have been investigated to develop novel kombucha beverages with distinctive features, not only in terms of their organoleptic properties but also with the aim of enhancing their nutritional and bioactive potential and diversity [13]. Although some commercial products are marketed as alternative or diverse because they include fruit juices, herbs, and similar ingredients, the main fermentation process still relies on tea leaves, and these additional components are generally incorporated after fermentation has been completed [15]. In contrast, many research efforts have focused on partially or completely replacing tea leaves as the substrate, fermenting these alternative raw materials with different SCOBYs (Table 1).

Among them, several herbal species of aromatic, culinary, and/or medicinal interest have been tested, including yarrow [16], liquorice, ginger [17], lemon balm [18], turmeric [19], winter savory, wild thyme, and purple basil [21]. Furthermore, other plants have also been explored; for instance, Reyes-Flores et al. (2023) fermented hempseed hearts together with tea leaves to increase antioxidant capacity, protein, and total phenolic content [22].

Interestingly, some specific plant parts have been targeted for the preparation of novel kombucha drinks. In this context, leaves from various trees, such as oak, quince, gingko biloba, and Indonesian bay, as well as from herbaceous species, such as peppermint, African mustard, and stinging nettle, have been utilized [20,23,24,25,26,27]. Even more than leaves, a wide variety of fruits have been fermented in experimental kombucha beverages, including cherry, plum, strawberry, strawberry tree fruit, apricot, persimmon, grape, orange, pomegranate, papaya, apple, black mulberry, rosehip fruit, Indian gooseberry, blueberry, passion fruit, and jujube [1,15,28,29,30,31,32,33,34,35,36,37,38]. Furthermore, flowers from elderberry and butterfly pea, as well as seeds such as Arabic coffee beans, were selected as raw materials [20,39,40,41], and, with the aim of revalorizing wastes from the fruit and vegetable industry, by-products from cocoa, coffee, guava, acerola, tamarind, and grape production were also exploited [42,43,44,45].

Apart from vegetal materials, fungal species such as mushrooms and truffles have been incorporated into specific kombucha beverages. In particular, reishi, turkey tail, and shiitake mushrooms, as well as black and summer truffles, have produced beverages with distinctive aromatic and compositional profiles [46,47,48].

Thus, a wide range of raw materials is being investigated as potential substrates for kombucha, leading to a great diversity of physicochemical, biochemical, and bioactive particularities. Logically, the differences in the nature of the substrate together with the composition of the microbial consortia and the fermentation matrix may influence the bioaccessibility and bioavailability of the bioactive compounds and, therefore, the biological properties of the kombuchas.

### 2.2. Physicochemical and Biochemical Characteristics of Alternative Kombucha Beverages

Both in traditional and alternative kombucha drinks, pH is expected to decrease during fermentation, mainly due to the production of acetic acid by AAB once ethanol is generated by yeasts from soluble sugars, but also as a result of the release of organic acids from the food matrix [15,49]. This trend was observed for all the innovative kombuchas described, although absolute values differed significantly depending on the selected substrate and, presumably, the nature of the SCOBY [8,13]. At the start of the fermentation process, the reported pH values ranged from approximately 3.0 to 7.0, registered for acerola by-product kombucha and African mustard leaf kombucha, respectively [25,44]. These significant differences can be explained by several factors. One might be the differential composition and content of organic acids in the substrates, as well as their solubility or releasability in the liquid medium. However, the main cause may be the use of a certain volume of the so-called ‘old kombucha’, which is the liquid where the SCOBY is immersed and kept before utilization. This addition of ‘old kombucha’ is usually carried out to lower the initial pH and prevent the growth of undesirable microorganisms, generally mold-like species, which are frequent contaminants in kombucha. For this reason, it is recommended to start fermentation at pH values below 4.2 [49]. Nevertheless, in many cases, kombucha beverages with higher initial pH values, such as the one prepared with acerola by-products, but also those made with turmeric (6.7), papaya (6.1), black and summer truffles (5.6–5.7), and butterfly pea flowers (5.4), did not show mold contamination or other undesirable microbes [19,29,39,44,48]. Depending on the fermentation conditions (time and temperature), SCOBY composition and activity, and, logically, the substrate utilized, the final pH values ranged from 2.0 for persimmon kombuchas to 4.0 for kombuchas prepared with citrus fruit and coffee residues, in both cases, after 21 days [15,43]. Ideally, kombucha drinks should be bottled at pH values above 2.5, since lower levels would suggest excessive acetic acid accumulation negatively affecting their organoleptic properties and constituting a risk to consumers’ health [49].

Regarding soluble sugars, the sucrose added at the beginning of the process is hydrolyzed into glucose and fructose by yeasts, which, in turn, produce ethanol. Therefore, the high concentrations of soluble sugars present at the initial stages of fermentation are drastically reduced by this alcoholic fermentation [7]. Sucrose is not the only source of carbohydrates in kombucha; the alternative raw material itself (or tea in the case of traditional kombucha) also provides carbohydrates with different solubilities in the liquid medium, as does the small volume of old kombucha added to the mixture [48].

Thus, the studies summarized in Table 1 reported their results in different ways: by determining the total soluble solids (in this type of measurement, the main contributors are sugars, namely, sucrose, glucose, and fructose, although alcohols, organic acids, or other minor compounds, such as phenolics, peptides, and minerals, may also interfere); by quantifying the total carbohydrates in the soluble fraction (after centrifugation, using well-established methods, such as the phenol–sulfuric acid assay); or by quantifying specific carbohydrates such as sucrose, glucose, or fructose, usually through chromatographic techniques [15,21,23].

The initial sugar content in kombucha is typically quite high, as large amounts of sucrose are added (commonly around 70 g/L) [15]. Although these are high values, in alternative kombuchas, this initial content varies considerably depending on the exact amount of sucrose added and the carbohydrates contributed by the raw material itself, the SCOBY, or the old kombucha. For instance, very low total soluble solid values have been reported for Arabic coffee kombucha (5.2%) [41], whereas very high values have been observed for butterfly pea flower kombucha (up to 25.3%) [39]. Nevertheless, the action of the SCOBY generally resulted in sugar levels that are nutritionally and health-wise desirable: at the bottling time, usually around 7 to 10 days, taking the bottling time of traditional tea kombucha as a reference, the sugar content is typically lower (<9–10%) than that of other beverages such as fruit juices or carbonated drinks, making it an appealing alternative [48]. Even when fermentation extends over many days (beyond the usual bottling period), the sugars are not completely consumed; a residual amount always remains that cannot be utilized or metabolized by the SCOBY. This can be observed in fruit-based kombuchas, which, after 21 days of fermentation, still contained between 1.4 and 1.5% (for strawberry tree fruit and grape kombucha) and 4.0% (for cherry kombucha) of total carbohydrates [15,28].

As previously mentioned, sugar fermentation by yeasts leads to ethanol production; therefore, depending on the bottling time, kombuchas may contain certain levels of ethanol. If these levels surpass the limit of 1.2% (*v*/*v*), these drinks must be labeled as alcoholic beverages and excluded from any nutritional or health claims [50]. However, ethanol content is normally reduced by the action of AAB, which oxidizes this molecule, generating acetic acid [49]. This trend of an initial increase followed by a subsequent decrease in alcoholic content throughout fermentation was observed not only for traditional but also for innovative kombuchas, leading to final products with low or negligible ethanol contents, although high values were reached on specific fermentation days in some cases. For instance, passionfruit kombucha showed ethanol concentrations of 6.2% after 10 days of fermentation, and shiitake kombuchas reached 4.3% on the third day of the process. In these cases, it is important to allow AAB to act in the beverages, reducing the ethanol content [47,51].

Moreover, this intense activity of AAB may lead to significant acetic acid production. As commented for pH, excessive acidity deteriorates the organoleptic properties of kombucha and constitutes a health risk for consumers due to alterations in dental and gastrointestinal physiology [52,53]. In the studied alternative kombuchas, the highest acetic acid content was reported for Indian gooseberry kombucha, reaching 46.7 g of acetic acid/L after 21 days [33]. For kombuchas and SCOBYs with excessively high acetic acid production, shorter fermentation periods are recommended while simultaneously controlling the ethanol content.

Among other compounds present in kombucha beverages, although these drinks are not considered high-protein products, the occurrence of specific proteins and peptides may be of interest for the potential functionality and bioactivity of kombuchas. Typically, traditional tea kombuchas contain around 3 µg of protein/mL [54], representing a low concentration compared with other macronutrients. However, innovative kombuchas have shown significantly higher contents, up to 31.0 and 36.4 µg of soluble proteins/mL in kombuchas fermenting black and summer truffles, respectively. These fungal-based kombuchas contained particularly higher levels (more than 10-fold) compared with other alternative kombuchas prepared with fruits in analogous processes and using similar SCOBYs [28,48]. The evolution of protein content throughout the fermentation process can show different trends depending on various phenomena. In some cases, the protein content increases as fermentation progresses due to microbial biomass growth, greater microbial protein production, and the release of soluble proteins and peptides from the matrix into the liquid medium. On the contrary, reductions can also be observed, particularly when pH is significantly low, leading to the denaturation and/or precipitation of protein structures [5,28,48,55].

A comparable effect can also be noticed for phenolic compounds, interesting molecules present in kombucha due to their well-studied biological activities, including antioxidant, anti-inflammatory, antimicrobial, and other activities. In this case, factors such as the acidic environment, microbial activity, and enzymatic release (hydrolysis, depolymerization, decomplexation, etc.) of phenolic molecules may increase the content of monomers and low-molecular-weight phenolics, thereby enhancing total phenolic compound counts through the most commonly used protocols. However, low pH, microbial degradation, and polymerization of phenolic monomers and oligomers can lead to the reductions observed in some kombuchas [15,30]. As shown in Table 1, the range of total phenolic content in alternative kombuchas is wide, from <1 mg/100 mL in the case of jujube kombucha to approximately 350 and 870 mg/100 mL in gingko biloba leaf and red grape kombuchas, respectively [26,30,38]. The great diversity within the alternative substrate groups makes it difficult to draw comparative conclusions on, for instance, whether fruit-based kombuchas result in lower or higher phenolic contents than those prepared with other raw materials.

### 2.3. Biological Activities of Alternative Kombucha Beverages

The composition of alternative kombuchas, highlighting their phenolic content but also considering the presence of other bioactive molecules, such as peptides, carbohydrates, vitamins, and others, positions these beverages as interesting products capable of exerting beneficial effects on human health. However, as shown in Table 2, most of the published studies followed in vitro protocols, with only a few using animal models, and no clinical trials to date, which hinders the extrapolation of the results to humans [11]. Moreover, the majority of publications have focused on phenolic compounds and antioxidant effects, underestimating the potential of other molecules and bioactivities.

#### 2.3.1. Antioxidant Activity

The term ‘oxidative stress’ refers to the physiological imbalance that occurs when the generation and accumulation of reactive oxygen species (ROS) exceed the capacity of cellular and tissue defense mechanisms to neutralize them. This condition can cause damage that contributes to the development of various diseases, including cancer, cardiovascular, respiratory, renal, and neurological disorders [57]. Because dietary antioxidants have proven effective in preventing such disturbances or reestablishing oxidative balance, plant-derived sources have been extensively studied in recent decades to isolate highly potent antioxidant compounds [58]. In this sense, traditional tea kombucha, as well as kombuchas prepared with alternative substrates, have been thoroughly investigated regarding their antioxidant potential (Table 2). In fact, the majority (87%) of publications focusing on the bioactivities of alternative kombuchas performed antioxidant activity analyses. Almost all of them used protocols based on radical-scavenging assays, correlating the presence and concentration of phenolic compounds with their ability to scavenge free radicals (DPPH^●^, ABTS^●+^, etc.) [15,18,32]. These biochemical methods provide rapid and valuable information but also have certain limitations, since the environment in which scavenging reactions occur is not representative of real physiological conditions in humans. Therefore, cellular and animal models constitute a more practical and appropriate approach. Nevertheless, only three works evaluating alternative kombuchas employed them for antioxidant studies. Vázquez-Cabral et al. (2017) used THP-1 human monocytic cells as a cellular model to assess the antioxidant effects of oak leaf kombuchas. These monocytes were differentiated into macrophages and induced to produce ROS by the addition of hydrogen peroxide. When the cells were treated with these kombuchas, oak (poly)phenols (catechin, gallocatechin, and phenolic acids were identified) effectively scavenged ROS and reduced the oxidative stress provoked by hydrogen peroxide [23]. Similarly, using oak leaf kombuchas, Gamboa-Gómez et al. (2017) validated their antioxidant effects in female C57BL/6 mice fed a high-saturated-fat and high-fructose diet to induce obesity and treated with oak leaf beverages for 3 months. Oxidative stress markers analyzed in collected blood plasma showed that supplementation with kombuchas fermented from the leaves of *Quercus convallata* and *Quercus arizonica* exerted in vivo antioxidant effects [24]. Moreover, Zubaidah et al. (2019) utilized another rodent model, Wistar rats with streptozotocin-induced diabetes. The animals were orally treated for 28 days with snake fruit kombucha, which improved oxidative stress markers (superoxide dismutase activities and malondialdehyde levels) [35].

As previously mentioned, researchers directly correlated antioxidant effects with plant/kombucha (poly)phenols. These secondary metabolites have been widely studied as antioxidant molecules, since their structure allows them to neutralize free radicals, chelate transition metals, and inhibit lipid oxidation [59]. Unfortunately, only a few studies mentioned other antioxidant compounds, such as DSL, a derivative of D-glucaric acid that is present in many plants and plant-based beverages, including alternative kombuchas, such as those prepared with Indian gooseberries [33]. Moreover, La Torre et al. (2024) attributed the antioxidant effects of jujube kombuchas not only to their phenolic content but also to the levels of certain vitamins (C and B_12_) [38].

#### 2.3.2. Immune-Modulatory Activity

Inflammatory reactions triggered by infection, injury, or irritation are non-specific immune processes that may become chronic and are associated with various diseases such as cardiovascular disorders, cancer, arthritis, and allergies. In this regard, a wide range of food sources, including plant-based beverages such as kombucha, have been examined to identify compounds with potential immune-modulatory or anti-inflammatory activities [60].

However, these properties remain underexplored in alternative kombuchas, and only three studies have been identified, all of them using in vitro procedures. In one of them, Barakat et al. (2024) evaluated the inhibitory activity of ethanolic extracts obtained from grape pomace kombucha against 15-lipooxigenase, obtaining promising results, particularly for beverages fermented at 20 °C [45]. Moreover, Vázquez-Cabral et al. (2017) used THP-1 human monocytes differentiated into macrophages as a cellular model to assess the immune-modulatory activity of oak leaf kombuchas. The macrophages were stimulated with lipopolysaccharide to induce inflammatory responses after treatment with the beverages, demonstrating that oak kombuchas were able to reduce the levels of pro-inflammatory cytokines such as IL-6 and TNF-α and to decrease nitric oxide (NO) production [23]. Mushroom-based kombuchas also exhibited interesting immune-modulatory applications. Sknepnek et al. (2021) obtained polysaccharide extracts from kombuchas prepared using turkey tail and shiitake mushrooms. These extracts were added to peripheral blood mononuclear cell (PBMC) cultures stimulated with phytohemagglutinin (PHA), leading to diverse immune-modulatory effects, with the reduction in Th2 cytokines and IL-10 secretion being the most prominent [47]. This significantly limited number of studies contrasts with the potential of the alternative raw materials used in fermentations. For instance, there is evidence that polysaccharides from *Ginkgo biloba* exhibited anti-inflammatory activity, as they reduced the secretion of inflammatory mediators in RAW264.7 cells [61]. It would be highly interesting to investigate whether the fermentation process modifies or even enhances the immune-modulatory activity.

#### 2.3.3. Antiproliferative/Antitumoral Activity

Cancer remains the second leading cause of mortality worldwide and is associated with substantial morbidity, representing a major global health concern. This has driven extensive research efforts aimed at identifying and validating novel approaches based on natural and edible products that may overcome the limitations of conventional anticancer drugs, including adverse side effects and low target specificity [62].

Again, only three in vitro studies were found on the antiproliferative activity of alternative kombuchas. Khazi et al. (2024) prepared kombuchas with different concentrations of turmeric and tested their cytotoxicity against A-431 human epidermoid squamous carcinoma cells. Interestingly, the effect was more potent in kombuchas with higher concentrations of turmeric, and the beverages fermented without turmeric did not show cytotoxicity against this cell line, confirming that the source of the antiproliferative compounds was this substrate [19]. Furthermore, Rahmani et al. (2019) evaluated the cytotoxic effect of different fractions obtained from African mustard leaf kombuchas against MCF-7 breast cancer cells. These fractions were obtained with different solvents (ethyl acetate, n-butanol, and water). Surprisingly, the fractions collected from unfermented infusions showed a significantly higher cytotoxic effect than those from fermented kombuchas, particularly the ethyl acetate one (approx. 45%). In fact, for the fermented samples, only the fraction extracted with n-butanol showed detectable but very low cytotoxicity (approx. 5%) [25]. In addition, kombuchas prepared with yarrow infusions and subcritical water extracts showed antiproliferative effects validated in different human tumoral cell lines, such as RD human rhabdomyosarcoma and Hep2c human cervix carcinoma-HeLa derivative cells [16]. Once again, the three studies largely attributed the biological effects of kombuchas to phenolic compounds. However, it would be of great interest to investigate the antitumor potential of other molecules, such as the organic acids released or produced during fermentation or the polysaccharides derived from the raw materials, as many of these have been shown to inhibit the growth of cancer cells or promote apoptosis [63].

#### 2.3.4. Hypoglycemic Activity

Diabetes is a chronic metabolic disorder primarily defined by hyperglycemia resulting from insufficient insulin production or impaired insulin sensitivity. While diabetic patients are typically treated with pharmacological interventions, such as metformin or insulin, alternative strategies based on functional dietary components may offer notable advantages by easing administration and reducing adverse side effects [64].

For alternative kombuchas, research efforts have mainly focused on demonstrating the in vitro ability of these beverages to inhibit key enzymes whose activity is linked to hyperglycemic effects, such as α-amylase and α-glycosidase. In this context, kombuchas prepared with mangrove and Indonesian bay leaf were capable of inhibiting α-glycosidase activity, while oak leaf and grape pomace kombuchas inhibited both enzymes [24,27,45,56]. However, unlike studies on other biological activities, hypoglycemic properties were also validated in animal models. The aforementioned oak leaf kombucha was administered for 3 months to female obese C57BL/6 mice fed a high-saturated-fat and high-fructose diet. After performing an oral glucose tolerance test, the treated animals showed lower glucose levels compared with the obesity control group and glucose tolerance similar to that of the healthy control group. Moreover, in a long-term evaluation, a reduction in fasting glucose concentrations was observed in treated animals [24]. Additionally, snake fruit kombuchas administered for 28 days to Wistar rats with streptozotocin-induced diabetes significantly reduced fasting plasma glucose levels. Furthermore, immunohistochemically staining of pancreatic tissues revealed an improvement in pancreatic β-cells in treated rats [35]. These hypoglycemic formulations showed significant levels of flavonoids, flavonoid glycosides, tannins, and saponins. However, deeper investigations to establish correlations between individual compounds and biological activities are still lacking.

#### 2.3.5. Antihypertensive and Hypolipidemic/Hypocholesterolemic Activity

Cardiovascular diseases are the leading cause of death worldwide, and high serum levels of total and LDL cholesterol, as well as hypertension, are considered major risk factors [65,66]. Despite this, only one study has investigated the antihypertensive activity, and another, the hypolipidemic activity, of alternative kombuchas. Regarding antihypertensive properties, as usual, the methodology involved an in vitro protocol measuring the inhibition of angiotensin-converting enzyme (ACE) activity. In this context, kombuchas prepared with different plant-based substrates (winter savory, wild thyme, peppermint leaves, stinging nettle leaves, quince leaves, and elderberry flowers) were reported as ACE inhibitors, showing a significantly higher capacity in stinging nettle and elderberry preparations [20]. Future perspectives should include additional studies not only demonstrating the ability of alternative kombuchas to inhibit ACE activity but also identifying the responsible proteins and peptides.

Moreover, the 28-day intervention with snake fruit kombucha in diabetic rats, described in previous sections, allowed the assessment of the effects of this beverage on lipid profiles. The animals treated with snake fruit kombucha showed reduced levels of triglycerides and total and LDL cholesterol, and increased levels of HDL cholesterol. Interestingly, this improvement was significantly more effective for snake fruit kombucha than with traditional black tea kombucha, highlighting the relevance of alternative and innovative substrates [35]. Surprisingly, although the substrates used can provide kombuchas with numerous molecules capable of exerting a hypolipidemic effect, for example, the carbohydrates present in the fiber of certain fruits [67], no further studies on this topic have been reported to date.

#### 2.3.6. Antimicrobial Activity

The rising prevalence of microorganisms that are resistant to conventional antimicrobial agents has intensified the search for new molecules capable of inhibiting or eradicating undesirable microbes. Many bioactive antimicrobial substances occur naturally in edible sources and have been investigated for their potential applications in food preservation as well as their activity against pathogenic species. Consequently, innovative strategies are essential to address antimicrobial resistance, including the discovery of newly isolated compounds, the exploration of molecules not previously assessed for antimicrobial action, and the redesign of natural products—leveraging their bioactive natural scaffolds—to generate more potent antimicrobial agents [68,69].

Traditional and alternative kombucha beverages have proven their antimicrobial capacity, demonstrating that this effect is not only linked to the acetic acid produced during fermentation and the inhibitory or microbicidal effect of certain SCOBY species, but also to the presence of bioactive molecules, such as phenolic compounds and vitamins. This antimicrobial activity has been reported for many innovative kombuchas prepared with turmeric, lemon balm, yarrow, coffee, fruits (black mulberry, black and red grape, rosehip fruit, and snake fruit), and reishi mushrooms [16,18,19,30,31,32,34,41,46]. The range of affected microorganisms is wide, including relevant food pathogens, such as strains of *Escherichia coli*, *Staphylococcus aureus*, *Salmonella* spp., *Bacillus cereus*, etc. Some of them also inhibited the growth of opportunistic pathogens, such as *Mucor racemosus* (kombuchas with apple, rosehip fruits, or black mulberry) and *Candida albicans* (kombuchas with yarrow or coffee) [16,31,32,41]. Moreover, other species of clinical relevance, such as bacteria related to urinary tract infections like *Staphylococcus saprophyticus* and *Citrobacter freundii*, were inhibited by lemon balm kombucha [18]. Arabic coffee kombucha showed an antifungal effect against *Cryptococcus gatti*, responsible for cryptococcosis, demonstrating the antimicrobial effects of alternative kombuchas that can be applied to health purposes beyond food safety [41].

### 2.4. Current Status of Alternative Kombucha Beverages Assessed Through a SWOT Analysis

**Figure 1:**
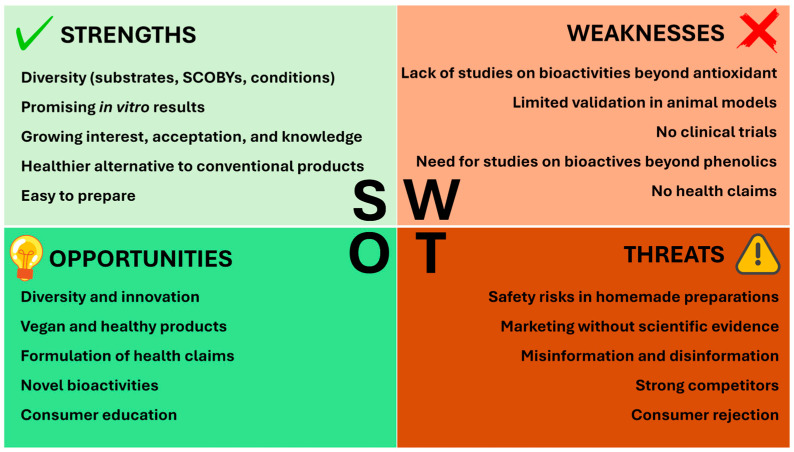
SWOT analysis of the current status of alternative kombucha beverages.

Considering the described evidence of alternative kombuchas as well as other factors, like consumers’ knowledge and perception or existing risks and threats, the SWOT methodology was applied to analyze the current status of alternative kombuchas (Figure 1).

#### 2.4.1. Strengths

An undoubted strength of alternative kombuchas is the great diversity of final products that can be reached by playing with different raw materials (other than *C. sinensis* leaves), SCOBYs with dissimilar microbial composition and activity, and conditions such as temperature, time, available oxygen, added sugar, etc. The possible combinations are huge and remain, logically, unexplored due to the existence of a plethora of substrates, fermentative microorganisms, and specific conditions [13].

Moreover, in vitro results regarding the biological activities of alternative kombuchas provide scientific evidence and motivate further investigation, since the reported effects are significantly promising (Table 2). Obviously, these capacities must be validated through animal models and clinical trials [11]. Another aspect that stimulates advances in novel kombucha research is the growing interest of consumers, particularly in Western countries, where kombuchas arrived substantially later, accompanied by an increased acceptance of these fermented products, and consumers are also more informed than in previous decades. However, exhaustive consumer studies must be carried out for a better understanding of their behavior nowadays [70]. Related to consumer acceptance, it is important to state again that, in terms of, for instance, sugar content, alternative kombuchas constitute a healthier beverage than others, such as carbonated drinks and juices, and, in some cases, even green and black tea kombucha [48]. This fact, together with the easy procedure to prepare kombucha that allows consumers to ferment their own homemade beverages, makes it an appealing product.

#### 2.4.2. Weaknesses

Although a considerable number of studies have already been carried out on the biological activities of alternative kombuchas, most of them were limited to antioxidant properties (Table 2). Moreover, almost the totality of the studies utilized in vitro assays, and only two works tested biological effects in vivo. Gamboa et al. (2017) demonstrated the antioxidant and hypoglycemic effect of oak leaf kombucha in obese mice, and Zubaidah et al. (2019) validated the antioxidant, hypoglycemic, and hypolipidemic/hypocholesterolemic effects of snake fruit kombucha in diabetic rats [24,35]. Despite some clinical trials evaluating conventional tea kombucha effects on humans having already been published, no clinical data related to alternative kombucha consumption can be found to date, hindering the extrapolation of the aforementioned promising results to human health. In addition, this lack of in vivo investigations limits the existence of data on the bioavailability of the target compounds. These obstacles explain the fact that there are no approved health claims for alternative (or traditional) kombuchas, so kombucha producers must not label their products with unclear messages suggesting the benefits of these beverages [71,72].

Another gap that can be identified as a weakness for alternative kombuchas is the lack of studies on bioactive molecules beyond phenolic compounds. The variety of substrates and sources deserves broadening the focus and paying attention to other molecules, such as proteins and peptides released during fermentation that may exert not only antioxidant but also antihypertensive activities, among others, as well as polysaccharides and other carbohydrates capable of modulating immune responses or exerting antiproliferative activity in tumoral cells, or even bioactive lipids that can be obtained from alternative matrices used to date, among other examples. Moreover, organic acids produced and released during fermentation can also be crucial for antitumoral and antimicrobial properties. In general, specific studies correlating individual compounds with particular biological activities need to be carried out [1,47].

#### 2.4.3. Opportunities

The diversity of ingredients used to prepare alternative kombuchas, which was previously mentioned as a strength, also constitutes an opportunity for researchers and producers to develop innovative products with unique characteristics. Moreover, the relatively late introduction of kombuchas to Western societies might be used as an advantage to attract consumers who are eager to access novel food products. These kombuchas, if derived from non-animal sources, will represent an appealing beverage for the vegan community, and although health claims have not been approved yet by authorities, the in vitro and in vivo evidence regarding their biological activities, along with their interesting nutritional profile, may motivate consumers to perceive kombuchas as a healthier alternative, at least in comparison with other beverages, such as carbonated drinks [48]. Additionally, the current lack of approved health claims should not be discouraging; on the contrary, it should encourage investigators and the industry to continue research and development until robust scientific evidence is generated, enabling authorities to eventually grant these claims [11].

Furthermore, new combinations of ingredients will likely open the door to previously unexplored bioactivities, extending the scope well beyond antioxidant activity. As an example, Rahmani et al. (2019) observed in vitro neuroprotective properties for African mustard leaf kombucha since it was able to inhibit acetylcholinesterase enzyme activity [25].

Finally, the existence of a consumer base interested in this type of product provides an opportunity for experts to take the initiative in promoting education and disseminating information to these consumers. By expanding their knowledge, it is possible that consumer behaviors will improve regarding the perception and consumption of alternative kombuchas, a product considered novel but that may currently be perceived as lacking robust evidence and clear information [71,72].

#### 2.4.4. Threats

Although the ease of preparation of alternative kombuchas has been previously mentioned as a strength, there are, indeed, certain risks associated with the production, handling, and storage of homemade kombuchas that may pose hazards to consumer health. For example, while the AAB in the SCOBY are expected to oxidize the ethanol produced by yeasts, reducing alcohol levels to negligible or very low concentrations, premature termination of fermentation may result in a final product with a significant alcohol content, making it unsuitable for specific populations, such as minors or pregnant individuals [49].

Conversely, excessively prolonged fermentation may lead to the accumulation of acetic acid at levels that are not only organoleptically unpleasant but may also pose potential health risks. Finally, the low pH at which fermentation occurs inhibits the growth of undesirable microorganisms, including pathogens; however, it is still recommended that individuals preparing kombucha at home maintain fermentation within a safe pH range (2.5–4.2) and adhere to basic hygiene practices during handling and storage [49].

One of the major threats to both traditional and alternative kombuchas is the dissemination of misleading messages by certain brands engaging in poor advertising practices, as well as by celebrities and social media influencers who share ambiguous statements, misinformation, and disinformation. All this must be countered with scientific evidence, and scientists and experts have a responsibility to combat false claims and provide accurate information to potential consumers. If messages are unclear and misinformation remains unchallenged, consumer rejection of these products may result, precisely due to contradictory information or the lack of evidence and its dissemination.

Furthermore, alternative kombuchas must face strong competitors in their target market. On the one hand, there are well-established traditional tea kombuchas with a firmly positioned market presence. On the other hand, widely consumed beverages over many years, such as juices, alcoholic, carbonated, and energy drinks, also compete for consumer attention [48].

## 3. Materials and Methods

### 3.1. Literature Search

A comprehensive literature search was conducted to identify scientific articles related to alternative kombuchas. The databases Web of Knowledge, Scopus, and PubMed were screened using a combination of general and specific keywords, including “kombucha”, “alternative kombucha”, “novel kombucha”, “fruit kombucha”, “herbal kombucha”, and “innovative kombucha”, among others. For studies reporting physicochemical parameters and biochemical composition, these terms were combined with concrete descriptors (“pH”, “carbohydrates”, “acetic acid”, etc.). To retrieve information on biological activities, additional keywords were included (“antioxidant”, “anti-inflammatory”, “hypocholesterolemic”, etc.). After exhaustive screening, only articles in which alternative kombucha was the main focus were selected. Experimental studies were used to construct the tables presented in Section 2, while review papers and studies on traditional kombucha were considered only as supporting sources.

### 3.2. SWOT Analysis

A critical evaluation of the current state of alternative kombucha research was carried out through a SWOT (Strengths, Weaknesses, Opportunities, and Threats) analysis following the methodology described by Puyt et al. (2023) [73]. A graphical matrix (Figure 1) was generated to summarize the key insights derived from the reviewed literature.

## 4. Conclusions

Kombuchas prepared from substrates other than tea leaves should be regarded as a novel beverage with nutritional, functional, and commercial interest. Their notable biochemical composition (lower sugar content compared with other beverages and presence of bioactive molecules, such as phenolic compounds, vitamins, and polysaccharides) and their promising in vitro results regarding biological activities represent a relevant strength, positioning them as attractive products for consumers, whose interest in this type of fermented beverage has increased in recent years. Furthermore, the tremendous diversity of substrates, SCOBY compositions, and fermentation conditions represents an important opportunity.

However, the field of alternative kombuchas must address current weaknesses, such as the scarcity of analyses on biological activities beyond antioxidant capacity, the lack of animal studies and clinical trials, and the limited evaluation of bioactive compounds other than phenolics, as well as the consequent absence of approved health claims. Additionally, combating misinformation and disinformation and pursuing robust scientific evidence is a responsibility for both researchers and industry. Several research advances must be encouraged: standardization of fermentation protocols across substrates, multi-omics approaches (metabolomics and metagenomics) to identify microbial–substrate interactions, in vivo evaluation of bioactives’ bioavailability and gut microbiota interactions, correlation studies between individual compounds and biological activities, and sensory analyses and consumer studies.

Only through these efforts can a solid base be established for the approval of health claims, ensuring the benefits of these beverages for human health, allowing legal and rigorous labeling to companies, and reinforcing consumer perception of them as safe products with scientifically demonstrated effects.

## Figures and Tables

**Table 1:** Physicochemical and biochemical characteristics of alternative kombucha beverages.

Alternative Substrate Group	Specific Substrate	pH	Total Soluble Solids (° Brix)	Total Carbohydrates (% *w*/*v*)	Ethanol (% *v*/*v*)	Soluble Proteins (µg/mL)	Acetic Acid (g/L)	Acidity (g/L)	Total Phenolic Compounds (mg/100 mL)	Reference
**Plants/herbs**	Yarrow (*Achillea millefolium*)	2.4–3.5	-	-	-	-	2–15 (approx.)	1.3–17.8	14–34	[16]
	Liquorice (*Glycyrrhiza uralensis*)	3.0–4.5 (approx.)	-	-	-	-	-	-	<10–61.9	[17]
	Ginger (*Zingiber officinale*)	3.2–5.2 (approx.)	-	-	-	-	-	-	<10–75.3	[17]
	Lemon balm (*Melissa officinalis*)	3.1–4.7	-	-	-	-	-	2.1–8.1	70.8–85.0	[18]
	Turmeric (*Curcuma longa*)	2.9–6.7	-	-	-	-	-	-	10–80	[19]
	Winter savory (*Satureja montana*)	<3–>4 (approx.)	-	-	-	-	0.18–0.48	<0.4–2 (approx.)	6–10	[20]
	Wild thyme (*Thymus serpyllum*)	<3–>4 (approx.)	-	-	-	-	0.41–0.60	<1–<2.5 (approx.)	9–11	[20]
	Purple basil (*Ocilum basilicum*)	3.0–3.1	7.1–8.5	-	-	-	-	8.0–8.4 (titratable)	22.5–26.6	[21]
	Hempseeds hearts (*Cannabis sativa sativa*)	-	-	-	-	0.3–7.8	-	-	10.5–88.1	[22]
**Leaves**	Oak leaves (*Quercus resinosa*, *Quercus arizonica*, and *Quercus convallata*)	2.8–3.5	-	Sucrose, glucose, and fructose were quantified	-	-	-	-	Individual species were identified and quantified	[23]
	Oak leaves (*Q. convallata* and *Q. arizonica*)	3.2–3.3	10.1–10.4	Sucrose, glucose, and fructose were quantified	-	-	-	66–68	Individual species were identified and quantified	[24]
	African mustard leaves (*Brassica tournefortii*)	3.0–7.0	-	Sucrose, glucose, and fructose were quantified	0.0–1.4	-	0–14	-	17.5–27.0 (mg/100 mg)	[25]
	Peppermint leaves (*Mentha piperita*)	<3–>5 (approx.)	-	-	-	-	0.12–1.94	0.1–4.5 (approx.)	10–15	[20]
	Stinging nettle leaves (*Urtica dioica*)	<3–4 (approx.)	-	-	-	-	0.18–3.03	<0.5–5 (approx.)	8–11	[20]
	Quince leaves (*Cydonia oblonga*)	3–5 (approx.)	-	-	-	-	0.14–2.39	<1–<2.5 (approx.)	11–12	[20]
	*Ginkgo biloba* leaves	2.6–3.8	10.7–17.0	-	-	-	-	0.2–1.8	480–870 (approx.)	[26]
	Indonesian bay leaf (*Syzygium polyanthum*)	2.9–3.1	-	-	-	-	-	-	-	[27]
**Fruits**	Summer (cherry: *Prunus avium*; plum: *Prunus domestica*; strawberry: *Fragaria* × *ananassa*; apricot: *Prunus armeniaca*) and winter (persimmon: *Diospyros kaki*; grape: *Vitis vinifera*; orange: *Citrus* × *sinensis*; pomegranate: *Punica granatum*) fruits	2.0–4.1	-	1.5–11.1	0.0–3.1	-	-	-	1.3–17.6	[15]
	Strawberry tree fruit (*Arbutus unedo*)	2.9–3.6	-	1.4–11.0	-	0.2–3.1	-	-	4.3–19.7	[28]
	Papaya (pulps and leaves) (*Carica papaya*)	2.8–6.1	7 (approx.)–14 (approx.)	-	0.0–1.2	-	0.0–1.6	-	-	[29]
	Red grape (*V. vinifera*)	2.9–4.0	-	-	0.0–0.9	-	-	25.9–104.2 (meq/L)	210–350 (approx.)	[30]
	Apple (*Malus domestica*)	3.0–3.5 (approx.)	-	Total sugars: 4–17 (approx.)	-	-	-	4–17	17.5–35 (approx.)	[31]
	Black mulberry (*Morus nigra*)	2.8–4.0	8.2–9.0	-	-	-	-	-	23.8–26.6	[32]
	Black grape (*Vitis lambrusca*)	2.5–3.5	6.9–7.6	-	-	-	-	-	14.3–16.0	[32]
	Rosehip fruit (*Rosa canina*)	2.6–3.3	7.1–7.8	-	-	-	-	-	6.7–7.2	[32]
	Indian gooseberry (*Phyllanthus emblica*)	2.2–4.0	5.3–13.0	-	0.0–2.1	-	6.7–46.7	-	5–70 (approx.)	[33]
	Snake fruit (*Salacca zalacca*)	-	-	-	-	-	-	4.4–16.5	27.5–62.3	[34]
	Snake fruit (*S. zalacca*)	3.2–3.9	12.9–13.9	-	-	-	-	5.7–15.6	28.1–53.6	[35]
	Blueberry (*Vaccinium myrtillus*)	3.1–3.5	-	-	-	-	-	-	96.3–116.7	[36]
	Passion fruit (*Passiflora edulis*)	3.2–3.5	4.0–13.1	-	6.2	300 (total protein)	-	11.3	13.2	[37]
	Apple (*Malus pumila*)	3.5–4.2	12.0–17.0	-	1.7	100 (total protein)	-	8.2	29.3	[37]
	Jujube (*Ziziphus jujuba*)	2.9–3.5	-	Sucrose, glucose, and fructose were quantified	0–1.4	1.8–5	0–31	-	0.9–1.2	[38]
**Flowers**	Elderberry flowers (*Sambucus nigra*)	<3–4 (approx.)	-	-	-	-	0.91–6.90	1–>15 (approx.)	8–12	[20]
	Butterfly pea flower (*Clitoria ternatea*)	2.5–5.4	13.0–25.3	Sucrose, glucose, and fructose were quantified	-	-	0–12.31	0–0.8	9–34	[39]
	Butterfly pea flower (*C. ternatea*)	3.5	-	-	0.2	-	1.65	-	129.4	[40]
Seeds/grains	Arabic coffee (*Coffea arabica*)	3.3–4.5	4.0–5.2	Reducing sugars: 3.9–5.1	-	-	-	0.8–7.2	51.1–57.1	[41]
**Vegetal by-products**	Cocoa bean shell (*Theobroma cacao*)	3.2–4.2	7–8 (approx.)	-	0			1–3 (mg/L, approx.)	9–24 (approx.)	[42]
	Citrus fruit residues and spent coffee grounds *(C. arabica*)	4.0–5.0	-	Cellulose production was measured	-	-	-	-	2.9	[43]
	Guava by-products (*Psidium guajava*)	2.9–3.5	7.3–8.1	-	-	-	7.3	2.6–7.5	-	[44]
	Acerola by-products (*Malpighia emarginata*)	2.6–3.0	6.6–7.5	-	-	-	14.7	3.2–9.4	-	[44]
	Tamarind by-products (*Tamarindus indica*)	2.8–3.2	7.4–8.3	-	-	-	5	4.4–10.0	-	[44]
	Grape pomace (*Vitis vinifera*)	2.9–3.4	2.1–5.3	Total sugars: 0.7–5.7	0.1–1.0	-	1.0–13.0	3.4–12.4	17.6–50.7	[45]
**Mushrooms**	Reishi mushroom (*Ganoderma lucidum*)	2.8–4.0	-	-	-	-	-	2.5–22.8	24.5	[46]
	Turkey tail mushroom (*Trametes versicolor*)	3.0–5.2	-	Total polysaccharides, sucrose, glucose, and fructose were quantified	0.0–3.1	-	-	1.0 (approx.)–33.5	19	[47]
	Shiitake mushroom (*Lentinula edodes*)	3.2–5.4	-	Total polysaccharides, sucrose, glucose, and fructose were quantified	0.0–4.3	-	-	1.0 (approx.)–23.4	33	[47]
**Truffles**	Black truffle (*Tuber melanosporum*)	2.5–5.6	-	2.5–7.4	0.0–1.6	7.5–31.0	-	-	1.8–50.7	[48]
	Summer truffle (*Tuber aestivum*)	2.8–5.7	-	1.8–6.4	0.0–0.7	4.0–36.4	-	-	4.3–64.4	[48]

**Table 2:** Biological activities of alternative kombucha beverages tested in different experimental models.

Biological Activity	Main Bioactive Compound/s	Alternative Substrate Group	Specific Substrate	Experimental Model/Methodology	Reference
**Antioxidant**	Phenolic compounds	Plants/herbs	Lemon balm (*M. officinalis*)	Radical-scavenging assays	[18]
			Turmeric (*C. longa*)	Radical-scavenging assays	[19]
			Ginger (*Z. officinale*)	Radical-scavenging assays	[17]
			Liquorice (*G. uralensis*)	Radical-scavenging assays	[17]
			Hempseed hearts (*C. sativa sativa*)	Radical-scavenging assays	[22]
		Leaves	Oak leaves (*Q. resinosa*, *Q. arizonica*, and *Q. convallata*)	THP-1 human monocytic cells	[23]
			Oak leaves (*Q. convallata* and *Q. arizonica*)	Radical-scavenging assays; C57BL/6 mice	[24]
			African mustard leaves (*B. tournefortii*)	Radical-scavenging assays	[25]
			*Ginkgo biloba* leaves	Radical-scavenging assays	[26]
		Fruits	Summer (cherry: *P. avium*; plum: *P. domestica*; strawberry: *Fragaria* x *ananassa*; apricot: *P. armeniaca*-) and winter (persimmon: *D. kaki*; grape: *V. vinifera*; orange: *Citrus* x *sinensis*; pomegranate: *P. granatum*-) fruits	Radical-scavenging assays	[15]
			Apple (*M. domestica*)	Radical-scavenging assays	[31]
			Black mulberry (*M. nigra*)	Radical-scavenging assays	[32]
			Black grape (*V. lambrusca*)	Radical-scavenging assays	
			Rosehip fruits (*R. canina*)	Radical-scavenging assays	
			Blueberry (*V. myrtillus*)	Radical-scavenging assays	[36]
			Snake fruit (*S. zalacca*)	Wistar rats	[35]
		Flowers	Butterfly pea flower (*C. ternatea*)	Radical-scavenging assays	[39]
		Seeds/grains	Arabic coffee (*C. arabica*)	Radical-scavenging assays	[41]
		By-products	Cocoa bean shell (*T. cacao*)	Radical-scavenging assays	[42]
			Citrus fruit residues and spent coffee grounds (*C. arabica*)	Radical-scavenging assays	[43]
	Phenolic compounds (flavonoids)	Plants/herbs	Purple basil (*O. basilicum*)	Radical-scavenging assays	[21]
			Winter savory (*S. montana*)	Radical-scavenging assays	[20]
			Wild thyme (*T. serpyllum*)	Radical-scavenging assays	
		Leaves	Peppermint leaves (*M. piperita*)	Radical-scavenging assays	
			Stinging nettle leaves (*U. dioica*)	Radical-scavenging assays	
			Quince leaves (*C. oblonga*)	Radical-scavenging assays	
		Flowers	Elderberry flowers (*S. nigra*)	Radical-scavenging assays	
		Mushrooms	Reishi mushroom (*G. lucidum*)	Radical-scavenging assays	[46]
	Phenolic compounds (flavonoids); DSL	Fruits	Indian gooseberry (*P. embilica*)	Radical-scavenging assays	[33]
	Phenolic compounds (flavonoids), ascorbic acid, and vitamin B12		Jujube (*Z. jujuba*)	Radical-scavenging assays	[38]
	Phenolic compounds (flavonoids and anthocyanins)	Flowers	Butterfly pea flower (*C. ternatea*)	Radical-scavenging assays	[40]
		Fruits	Red grape (*V. vinifera*)	Radical-scavenging assays	[30]
			Grape pomace (*V. vinifera*)	Radical-scavenging assays	[45]
	Phenolic compounds and organic acids		Snake fruit (*S. zalacca*)	Radical-scavenging assays	[34]
	-	By-products	Guava by-products (*P. guajava*)	Radical-scavenging assays	[44]
			Acerola by-products (*M. emarginata*)	Radical-scavenging assays	
			Tamarind by-products (*T. indica*)	Radical-scavenging assays	
Immune-modulatory	Phenolic compounds	Leaves	Oak leaves (*Q. resinosa*, *Q. arizonica*, and *Q. convallata*)	THP-1 human monocytic cells	[23]
	Phenolic compounds (anthocyanins)	Fruits	Grape pomace (*V. vinifera*)	5-lipoxygenase inhibition assay	[45]
	Polysaccharides and phenolic compounds	Mushrooms	Turkey tail mushroom (*T. versicolor*)	PBMCs	[47]
			Shiitake mushroom (*L. edodes*)	PBMCs	[47]
Antiproliferative/antitumoral	Phenolic compounds	Plants/herbs	Turmeric (*C. longa*)	A-431 cells	[19]
		Leaves	African mustard leaves (*B. tournefortii*)	MCF-7 cells	[25]
	Phenolic compounds and vitamin C	Plants/herbs	Yarrow (*A. millefolium*)	RD and Hep2c cells	[16]
Hypoglycemic	Phenolic compounds	Leaves	Oak leaves (*Q. convallata* and *Q. arizonica*)	α-amylase and α-glycosidase inhibition assays, glucose diffusion assay, and C57BL/6 mice	[24]
		Fruits	Snake fruit (*S. zalacca*)	Wistar rats	[35]
	Phenolic compounds (flavonoids and tannins) and saponins	Leaves	Indonesian bay leaf (*S. polyanthum*)	α-glycosidase inhibition assays	[27]
	Phenolic compounds (flavonoid glycosides)	Leaves	Mangrove leaves (*Rhizophora mucronata*)	α-glycosidase inhibition assays	[56]
	Phenolic compounds (anthocyanins)	Fruits	Grape pomace (*V. vinifera*)	α-amylase and α-glycosidase inhibition assays	[45]
Antihypertensive	Phenolic compounds (flavonoids)	Plants/herbs	Winter savory (*S. montana*)	ACE inhibition assay	[20]
			Wild thyme (*T. serpyllum*)	ACE inhibition assay	
		Leaves	Peppermint leaves (*M. piperita*)	ACE inhibition assay	
			Stinging nettle leaves (*U. dioica*)	ACE inhibition assay	
			Quince leaves (*C. oblonga*)	ACE inhibition assay	
		Flowers	Elderberry flowers (*S. nigra*)	ACE inhibition assay	
Hypolipidemic/hypocholesterolemic	Phenolic compounds	Fruits	Snake fruit (*S. zalacca*)	Wistar rats	[35]
Antimicrobial	Phenolic compounds	Plants/herbs	Turmeric (*C. longa*)	Microbiological analyses	[19]
			Lemon balm (*M. officinalis*)		[18]
		Fruits	Apple (*M. domestica*)		[31]
			Black mulberry (*M. nigra*)		[32]
			Black grape (*V. lambrusca*)		
			Rosehip fruits (*R. canina*)		
	Phenolic compounds and organic acids	Fruits	Snake fruit (*S. zalacca*)		[34]
	Phenolic compounds	Seeds/grains	Arabic coffee (*C. arabica*)		[41]
	Phenolic compounds (flavonoids)	Mushrooms	Reishi mushroom (*G. lucidum*)		[46]
	Phenolic compounds (anthocyanins)	Fruits	Red grape (*V. vinifera*)		[30]
	Phenolic compounds and vitamin C	Plants/herbs	Yarrow (*A. millefolium*)		[16]
Neuroprotective	Phenolic compounds	Leaves	African mustard leaves (*B. tournefortii*)	Acetylcholinesterase inhibition assay	[25]

## Data Availability

No new data were created or analyzed in this study. Data sharing is not applicable to this article.

## Abbreviations

The following abbreviations were used in this manuscript:

SCOBY	Symbiotic consortium/culture of bacteria and yeasts
SWOT	Strengths, Weaknesses, Opportunities, and Threats
AAB	Acetic acid bacteria
LAB	Lactic acid bacteria
DSL	D-saccharide-1,4-lactone
ROS	Reactive oxygen species
